# Effect of Eu^3+^ ion concentration on optical and magnetic properties of oriented Gd_2_O_3_/CTAB nanoparticles as multifunctional optical-magnetic probes in biomedicine

**DOI:** 10.1039/d5ra00932d

**Published:** 2025-03-28

**Authors:** Thi Lien Pham, Cong Quang Tong, Ngoc Phan Vu, Thi Hong Ha Vu, Thi Anh Ho, Duc Thang Pham, Thi Hoi Le, Manh Tien Dinh, Thanh Huong Nguyen, Thi Khuyen Hoang, Thi Kieu Giang Lam, Vu Nguyen, Hong Nam Pham, Tien Ha Le

**Affiliations:** a Institute of Materials Science, Vietnam Academy of Science and Technology 18 Hoang Quoc Viet, Cau Giay Hanoi Vietnam; b Faculty of Biotechnology, Chemistry and Environmental Engineering, Phenikaa University Hanoi 12116 Vietnam ha.vuthihong@phenikaa-uni.edu.vn; c Faculty of Engineering Physics and Nanotechnology, VNU University of Engineering and Technology, Vietnam National University, Hanoi 144 Xuan Thuy, Cau Giay Hanoi 11310 Vietnam; d Faculty of Physics, VNU University of Science, Vietnam National University, Hanoi 334 Nguyen Trai, Thanh Xuan Hanoi 11416 Vietnam; e Hanoi Medical University 01 Ton That Tung, Dong Da Hanoi Vietnam; f Institute of Science and Technology, TNU-University of Sciences Thai Nguyen 250000 Vietnam letienha@tnu.edu.vn

## Abstract

The Gd_2_O_3_:Eu^3+^ nanoparticles were synthesized using a multi-step chemical method with urea as a reactant to control the ratio of different Eu^3+^ activation centers: 2, 4, 6, 8, 10, and 12 mol% combined with CTAB surfactant to improve surface quality. The study aimed to determine the optimal concentration of Eu^3+^ in the presence of CTAB to increase biocompatibility and achieve the best fluorescence. The structure, surface morphology, optical properties, and magnetic properties of the materials were analyzed through FSEM, XRD, HRTEM, XPS, UV-vis, fluorescence, fluorescence excitation, time-resolved fluorescence, vibrating sample magnetometry (VSM), and magnetic heating measurements. The obtained material had a diameter of 180–280 nm, and it emitted red light with characteristic shifts from ^5^D_0_ to ^7^F_*J*_ (*J* = 0–4). The strongest emission peak occurred at the transition of ^5^D_0_ to ^7^F_2_, corresponding to a wavelength of 611 nm. The crystal is in the cubic phase. The highest lifetime of the samples is 2.1 ms, and the highest calculated quantum efficiency is 91% for the Gd_2_O_3_:8% Eu^3+^ sample. The M–H hysteresis curve revealed that the highest magnetic field obtained was 1.83 emu g^−1^. Experimental induction heating of samples reached temperatures in the range of 44–49 °C, which is an appropriate temperature range for destroying cancer cells without affecting healthy cells. These findings demonstrate that the material has great potential in cancer diagnosis and treatment.

## Abstract 2

The Gd_2_O_3_:Eu^3+^ nanoparticles were synthesized using a multi-step chemical method with urea as a reactant to control the ratio of different Eu^3+^ activation centers: 2, 4, 6, 8, 10, and 12 mol% combined with CTAB surfactant to improve surface quality.

## Graphical abstract

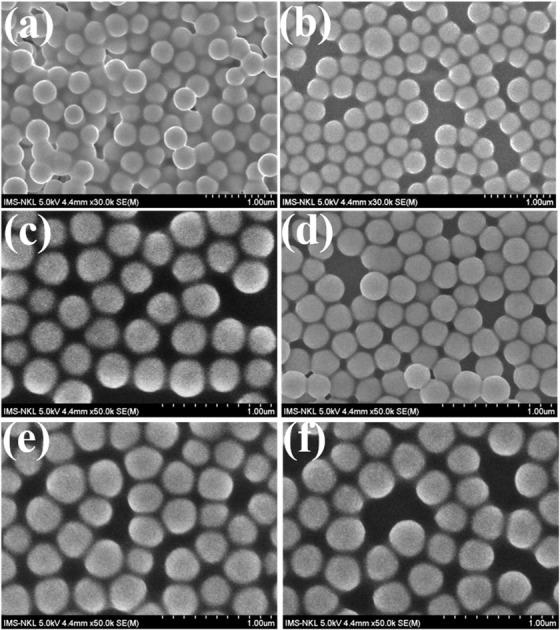

## 1. Introduction

Rare earth elements (RE) have garnered significant interest in both basic and applied research over recent decades due to their unique physical and chemical properties.^1–4^ This interest is reflected in the growing number of applications, as RE elements have become indispensable for important technologies.^5–7^ Nanostructured materials containing RE elements, either as the main component or dopant phase, have opened up new avenues for various biomedical applications, including bioimaging, biosensors, targeted drug delivery, and other therapies.^8–11^

The fluorescence properties of inorganic fluorescent materials depend closely on their substrate, dopant ions, size, and morphology.^12^ Researchers have thus explored the synthesis of inorganic luminescent materials with different substrates, dopant ions, and shapes.^12,13^ The choice of substrate significantly impacts the luminescent properties of materials, making substrate exploration a focal point for researchers.

Gd_2_O_3_ (gadolinium sesquioxide) is a well-known material widely used in cathode ray tubes, phosphors, bioimaging, and biosensors.^1–3^ Among various rare earth oxide materials, Gd_2_O_3_ offers several advantages, including physical, chemical, and thermal stability, low phonon energy, high refractive index, high dielectric constant, paramagnetism, and high density.^10,14,15^ When doped with europium (Eu^3+^), Gd_2_O_3_ becomes a red luminescent material with potential applications in fluorescent lamps, white light-emitting diodes, plasma display panels, flat screens, cathode ray tubes, MRI contrast agents, biosensors, and bioimaging.^16^ The long excitation level of Gd^3+^ ions produces emission lines in the UV region, and luminescence changes occur when other rare earth ions are added.^17^ Importantly, the ionic radius of Eu^3+^ matches that of Gd^3+^, allowing easy incorporation into the Gd_2_O_3_ substrate without distorting the crystal structure.^11,18^

On the other hand, gadolinium (Gd) and Gd^3+^ ions possess seven unpaired electrons, resulting in strong superparamagnetic properties.^19^ Organic hybrid compounds containing Gd^3+^ ions are commonly used as contrast agents in magnetic resonance imaging (MRI).^20–22^ In modern medicine, there is a growing emphasis on combining diagnosis and treatment.^23^ Therapies such as targeted drug delivery, chemotherapy, thermotherapy, or radiotherapy are increasingly integrated with imaging diagnostics, both *in vitro* and *in vivo*. Various imaging methods, including MRI and fluorescent labeling, can be combined with treatment agents like thermotherapy, chemotherapy, or drug delivery. This combined imaging approach allows for better control, information gathering, and understanding of process dynamics, ultimately enhancing treatment effectiveness.

In our study, we focus on evaluating the luminescent properties of Gd_2_O_3_ material at different Eu^3+^ doping concentrations, while also investigating the magnetic properties and thermotherapy potential of Gd_2_O_3_:Eu^3+^. Luminescent Gd_2_O_3_:Eu^3+^ materials have been synthesized using various methods, such as sol–gel, hydrothermal, co-precipitation, and multi-step chemistry.^11,24–26^ Among these methods, the multi-step chemical synthesis stands out as an easy and cost-effective approach to produce uniform-sized nanoparticles in a shorter time and at lower temperatures. Therefore, we will use this method to synthesize Gd_2_O_3_:Eu^3+^ material combined with CTAB surfactant to enhance surface quality. Using CTAB not only improves the dispersion of nanoparticles but also enhances their stability in biological environments. This research can lead to improved biocompatibility, making the nanoparticles more effective for *in vivo* applications. Investigating the relationship between optical properties and hyperthermia allows for the design of nanoparticles with tunable heating profiles. This can optimize treatment protocols, providing controlled thermal doses to target tissues while minimizing damage to surrounding healthy cells. The novelty of this research lies in the intersection of improved optical properties, enhanced stability through CTAB, and the potential for real-time imaging during hyperthermia treatments.

## 2. Materials and methods

### 2.1. Materials

Chemicals used in material synthesis Gd_2_O_3_:Eu^3+^: Gd(NO_3_)_3_·6H_2_O from Sigma-Aldrich, 99.9%, Eu(NO_3_)_3_·5H_2_O from Sigma-Aldrich 99.9%, urea (CO(NH_2_)_2_) from Sigma-Aldrich, hexadecyltrimethylammonium bromide (CTAB) from Sigma-Aldrich.

### 2.2. Synthesis process

Prepare “Solution 1” by mixing 0.05 M Eu(NO_3_)_3_·5H_2_O and 0.05 M Gd(NO_3_)_3_·6H_2_O in a 100 mL beaker, adjusting the Eu^3+^ : Gd^3+^ ratio to 2%, 4%, 6%, 8%, 10%, or 12%.

In a 500 mL flask, prepare a 0.5 M urea solution and gradually add “Solution 1,” stirring for 2 hours. Add 0.01 M CTAB and heat the mixture to 85 °C for 70 minutes to produce a white precipitate of Gd(OH)CO_3_·H_2_O.

**Scheme 1:**
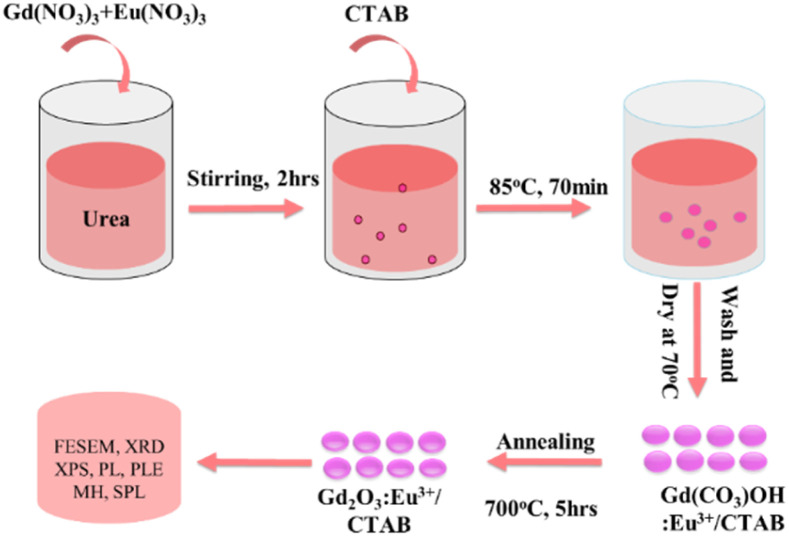
Schematic diagram of preparation process of the Gd_2_O_3_:Eu^3+^/CTAB.

Centrifuge the precipitate with deionized water and ethanol, then dry at 70 °C for 24 hours. Finally, calcine the material at 700 °C for 5 hours (Scheme 1).

### 2.3. Characterization techniques

The crystal structure of the nanoparticles was analyzed using X-ray diffraction (XRD) with a Bruker D8 Advance instrument with CuKα radiation (*λ* = 0.154 nm) at fine steps of 0.02°. The morphology of the synthesized material was analyzed using Field Emission Scanning Electron Microscopy (FESEM) on a Hitachi S-4800 machine. High-resolution transmission electron microscopy (HR-TEM) spectra were measured by a JEM2100 system (Jeol, Japan). X-ray photoelectron spectroscopy (XPS, Nexsa G2) analyzed the chemical bonding configurations. The bandgap energy was estimated using the UV-vis absorption spectrum obtained by JASCO V-750 spectrophotometer. Photoluminescence (PL) spectra were recorded with a Nanolog spectrophotometer (Horiba Jobin Yvon) excited by a 450 W xenon lamp. The saturation magnetization (MS) values were characterized by a vibrating sample magnetometer (VSM, MicroSense EZ9). The inductive heating experiment was conducted using an RDO-HFI device with an output power of 5 kW. All measurements were done in ambient air.

## 3. Results and discussion

### 3.1. Characterization of the Gd_2_O_3_ nanoparticles

#### 3.1.1. Morphology

**Fig. 1:**
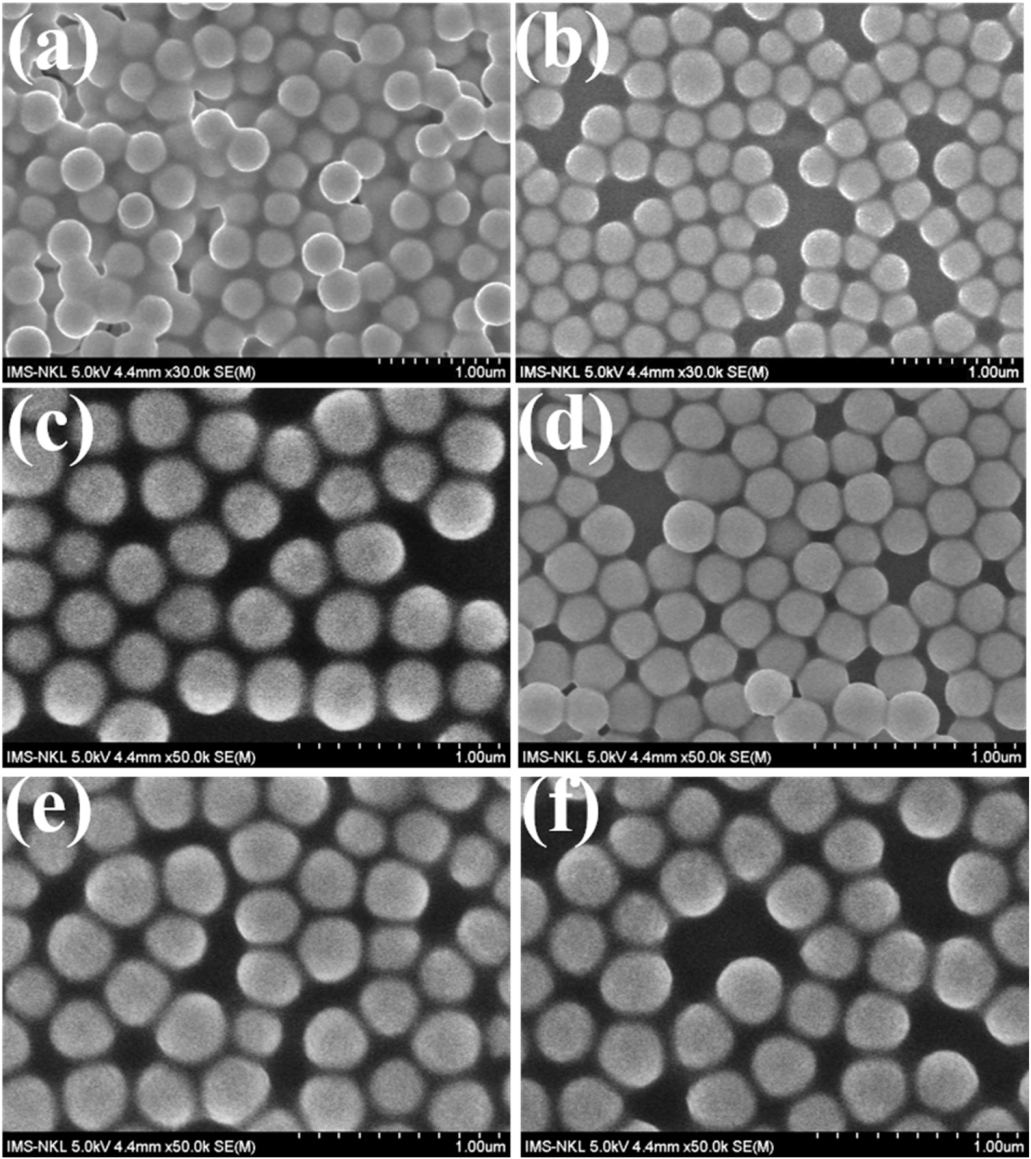
FESEM images of Eu-doped Gd_2_O_3_/CTAB with concentrations of 2% (a), 4% (b), 6% (c), 8% (d), 10% (e) and 12% (f).

When Eu^3+^ is doped at varying molar ratios of Eu^3+^/Gd^3+^ (from 2% to 12%) in combination with CTAB, uniform spherical nanoparticles are produced. At a low Eu^3+^ doping concentration of 2% (Fig. 1a), the resulting nano-spheres have a diameter of 180–200 nm. Increasing the Eu^3+^ concentration to 4% (Fig. 1b) results in nano-spheres measuring 200–220 nm (Fig. 1c). At a doping level of 6% (Fig. 1c), the nano-spheres expand to diameters of 210–230 nm. With 8% Eu^3+^, the spheres measure 220–230 nm (Fig. 1d), while a 10% doping level produces spheres with diameters of 230–240 nm (Fig. 1e). Notably, even at a high doping level of 12% (Fig. 1f), the synthesized spheres retain their spherical shape, with diameters ranging from 220–280 nm.

These results indicate that variations in the Eu^3+^ doping ratio significantly affect both the shape and size of the material, with a clear trend of increasing size as the Eu^3+^ doping ratio rises.

#### 3.1.2. XRD patterns

**Fig. 2:**
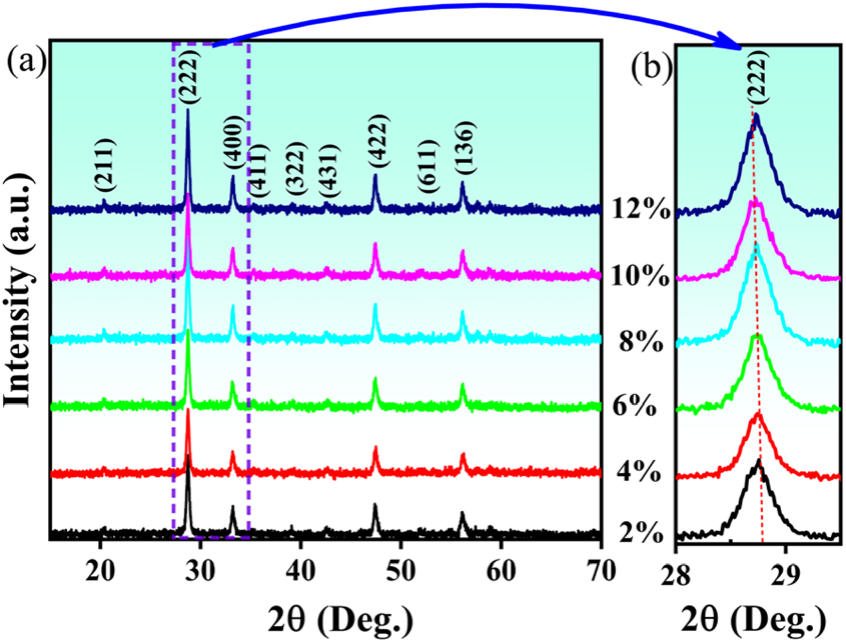
X-ray diffraction patterns of Eu-doped Gd_2_O_3_/CTAB samples with concentrations ranging from 2 to 12% in the range of 15 to 70 degrees (a) and magnification of the (222) peak (b).

The X-ray diffraction patterns of Eu^3+^-doped Gd_2_O_3_/CTAB nanoparticles with Eu^3+^ ion content ranging from 2 to 12% in Fig. 2 show distinct diffraction peaks at 2*θ* values of 20.12°, 28.59°, 33.13°, 38.94°, 42.51°, 47.42°, and 56.29°. These peaks correspond to the crystal planes (211), (222), (400), (332), (431), (422), and (662), which are characteristic cubic structures of Gd_2_O_3_, as indicated by the XRD reference pattern (JCPDS card 00-012-0797).^12^ The magnification results from 28 to 29.5° with the (222) lattice plane show that the position of this diffraction peak almost does not change when the Eu concentration increases, which shows that when Eu replaces Gd in the Gd_2_O_3_ cubic lattice, it does not distort the crystal lattice of the material. This result is believed to be due to the Eu^3+^ ion radius being similar to the Gd^3+^ radius, so the replacement process does not greatly affect the material's crystal lattice. At the same time, the X-ray diffraction pattern of the Gd_2_O_3_/CTAB material samples doped with Eu^3+^ ions does not show any new X-ray diffraction peaks (especially the diffraction peak of the Eu_2_O_3_ structural phase) when the Eu concentration changes from 2 to 12%. Based on the X-ray diffraction pattern, we have determined the average crystallite size of the Eu-doped Gd_2_O_3_/CTAB nanoparticles with different concentrations using the Scherrer formula eqn (1):1
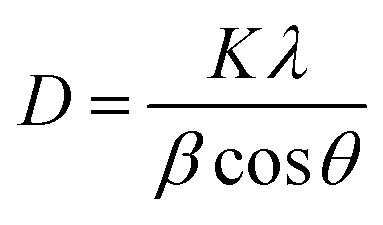
where *D* is the average crystallite size, *K* is the dimensionless constant value (0.89), *λ* = 1.540 Å is the wavelength of the irradiation and *β* is the full width at half maximum measured in radian. Calculating according to eqn (1), we obtained the average crystallite sizes of the materials as 25.40, 27.73, 27.04, 26.93, 26.43 and 26.93 nm corresponding to the Eu-doped Gd_2_O_3_/CTAB samples with concentrations of 2, 4, 6, 8, 10 and 12%, respectively.

#### 3.1.3. High-resolution transmission electron microscopy

**Fig. 3:**
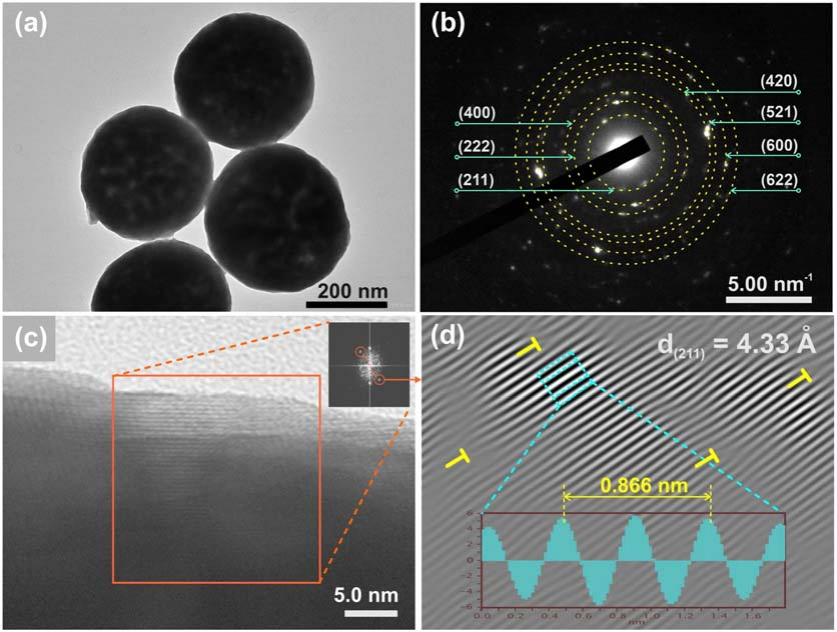
(a) TEM, (b) electron diffraction, (c and d) HRTEM images of Gd_2_O_3_/CTAB sample doped with 8% Eu^3+^ ions.

To determine the structure of Eu-doped Gd_2_O_3_/CTAB material more precisely, we measured the HRTEM of Gd_2_O_3_/CTAB sample doped with 8% Eu^3+^ ions and obtained the results in Fig. 3. The results observed in Fig. 3a show that the particles have an average diameter of about 280 nm with a typical cubic structure of Gd_2_O_3_ material with characteristic lattice planes (211), (222), (400), (420), (521), (600), and (662) (Fig. 3b) with the distance *d* between the lattice planes (211) shown in Fig. 3b is 3.44 Å. The HRTEM analysis results are consistent with the XRD results, confirming the consistency in the structure analysis by HRTEM and XRD.

#### 3.1.4. XPS spectra

**Fig. 4:**
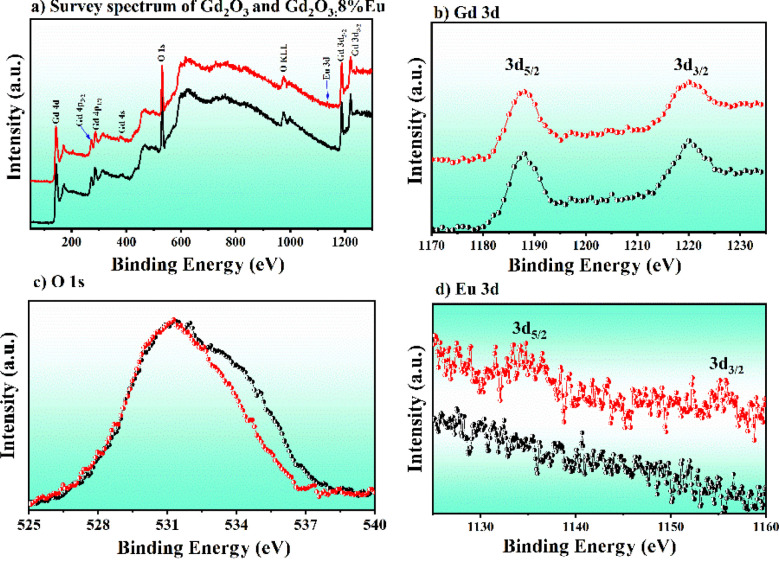
XPS spectrum of Gd_2_O_3_ and Gd_2_O_3_:8% Eu nanorods: (a) survey spectrum, (b) Gd 3d, (c) O 1s and (d) Eu 3d levels.

To further determine the chemical bonding state of Gd_2_O_3_/CTAB and Gd_2_O_3_:Eu^3+^/CTAB materials, we conducted XPS measurements of Gd_2_O_3_/CTAB and Gd_2_O_3_:Eu^3+^/CTAB samples doped with 8% Eu^3+^ ions. Fig. 4 shows the XPS spectra of two samples, Gd_2_O_3_/CTAB and Gd_2_O_3_:Eu^3+^/CTAB, doped with 8% Eu^3+^ ions, Gd 3d (b), O 1s (c) and Eu 3d (d). The measuring device corrected the survey data by C 1s (284.7 eV). Fig. 4a shows the characteristic peaks of Gd, O in both Gd_2_O_3_/CTAB and Gd_2_O_3_:Eu^3+^/CTAB samples. However, with the Gd_2_O_3_/CTAB sample supplemented with Eu element, the Eu 3d peak is located at about 1135 eV. For the Gd_2_O_3_/CTAB sample, in the high energy region of the 3d state of the Gd^3+^ ion, there are two characteristic Gd 3d binding peaks at the positions 1188.11 eV and 1219.98 eV (ref. 27) with a separation of 31.87 eV, indicating the presence of Gd 3d_5/2_ and Gd 3d_3/2_. However, in Fig. 4b, it is observed that these two Gd 3d peaks are almost insignificantly shifted in both the Gd_2_O_3_/CTAB and Gd_2_O_3_:Eu^3+^/CTAB samples.

**Fig. 5:**
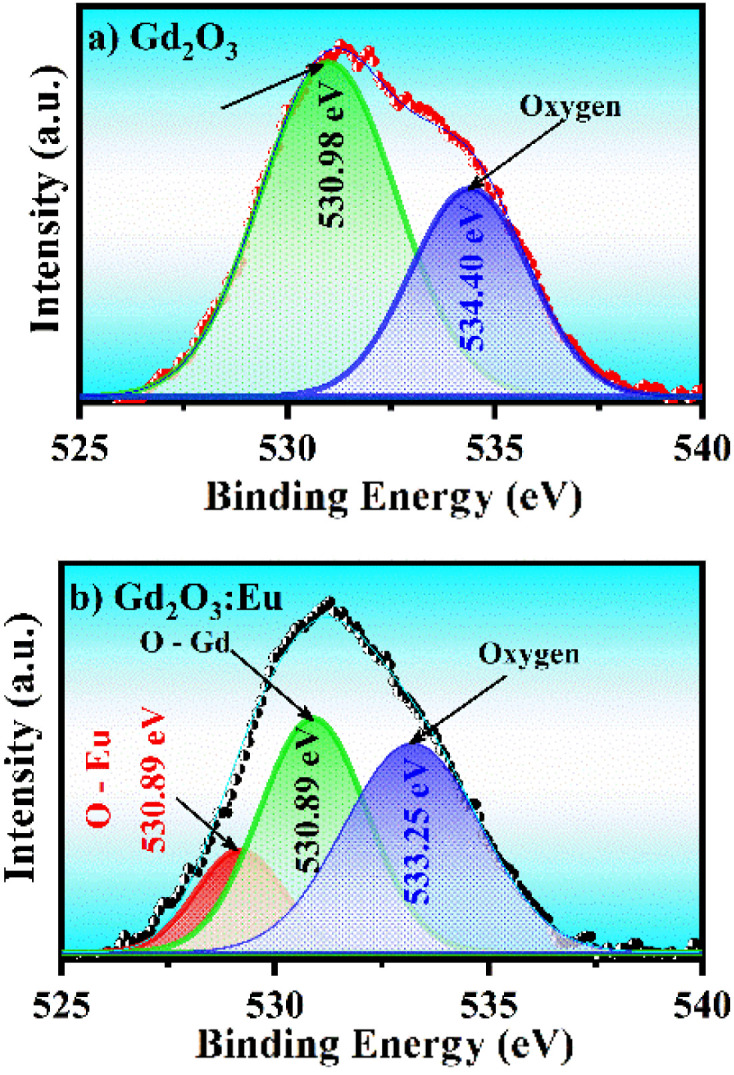
High-resolution XPS spectra of Gd_2_O_3_ (a) and Gd_2_O_3_:8% Eu (b) nanoparticles corresponding to the deconvoluted O 1s level.

However, the half-width of the peak was broadened, proving that when Eu was doped into the Gd_2_O_3_ lattice, it affected the local crystal field at the position where the Eu^3+^ ion replaced the Gd^3+^ ion. The effect of this substitution also changed the bonding of the O atom with other ions. Fig. 4c shows the bonding state of O 1s, and the result shows that when there is a circular shoulder of the Eu^3+^ ion in the lattice, the half-width of the O 1s peak narrows towards low energy. This result is believed to be due to the Eu^3+^ ion radius and its electronegativity (1.0) being smaller than the Gd^3+^ ion radius and electronegativity (1.2), narrowing the half-width towards this low binding energy region. To compare this phenomenon, we conducted a survey of the high-resolution XPS spectrum of the O 1s state and fitted the peaks corresponding to the characteristic bonds in Fig. 5. At the same time, the high-resolution XPS spectrum in the range from 1125 eV to 1160 eV in Fig. 4d shows that in the Gd_2_O_3_:Eu^3+^/CTAB sample, peaks appeared at 1134.25 eV and 1155.15 eV corresponding to the Eu 3d_5/2_ and Eu 3d_3/2_ states with a separation of 20.9 eV of the Eu^3+^ ion, while in the Gd_2_O_3_/CTAB sample, these two bond peaks were not present.

### 3.2. Optical studies

**Fig. 6:**
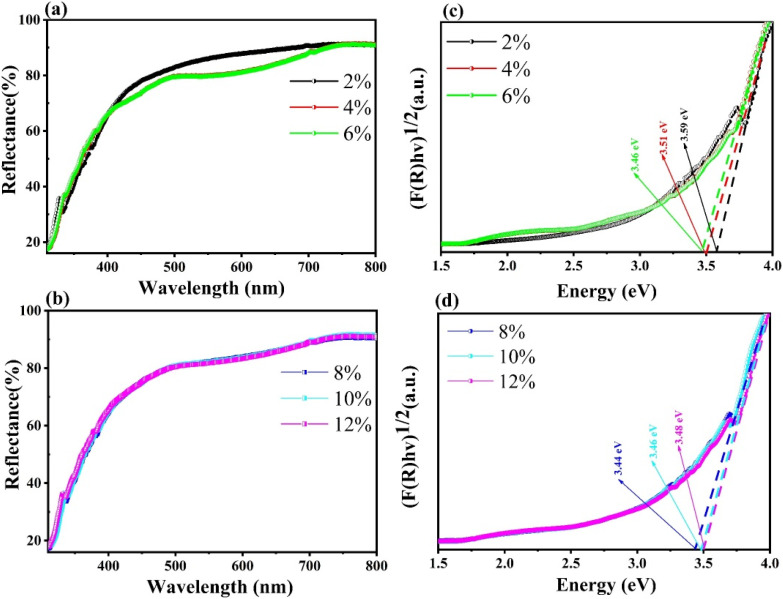
UV-vis spectra of Gd_2_O_3_:Eu^3+^ with different mol concentrations (a and b) and energy band gap of Gd_2_O_3_:Eu^3+^ for different dopant concentrations (c and d) using K–M theory.

In this study, the band gap of Gd_2_O_3_/CTAB and Gd_2_O_3_:Eu^3+^/CTAB materials was deduced from the UV-vis spectrum according to the Kubelka–Munk eqn (2):

2 (*F*(*R*_∞_)*hν*)^*γ*^ = *A*(*hν* − *E*_g_)

where (*F*(*R*_∞_)*hν*)^*γ*^ and *hν* are the reflection coefficient and photon energy, *R*_∞_ in the % reflectance obtained. *A* is a characteristic constant of the specific material, respectively. The value of *γ* is dependent on the origin of transition in a semiconductor (*γ* = 1/2 and 2 for indirect transition, respectively and allowed direct transition). In our case, *γ* = 1/2 because Gd_2_O_3_/CTAB and Gd_2_O_3_:Eu^3+^/CTAB are indirect transition semiconductors. Hence, *E*_g_ value of all samples could be calculated by plotting (*F*(*R*_∞_)*hν*)^1/2^*versus hν*, as illustrated in Fig. 6. The calculated band gap energies of Gd_2_O_3_/CTAB and Gd_2_O_3_:Eu^3+^/CTAB in Fig. 6 for samples with Eu^3+^ ion concentrations of 2, 4, 6, 8, 10 and 12% are 3.59, 3.51, 3.46, 3.44, 3.46, and 3.48 eV respectively. This result shows that when the concentration of Eu^3+^ ions doped into the matrix increases, the band gap of the Gd_2_O_3_ material decreases, and the band gap of the Eu-doped Gd_2_O_3_/CTAB material reaches its smallest value at a doping concentration of 8%. When the concentration increases above 8%, the material's band gap tends to increase. The result of this phenomenon is that when the doping concentration increases above a certain threshold, these doping ions cluster together and form clusters and escape from the matrix, leading to a decrease in the density of Eu^3+^ ion emission centers when the doping concentration increases above 8%. This phenomenon will affect the optical properties of the material.

### 3.3. Optical properties

**Fig. 7:**
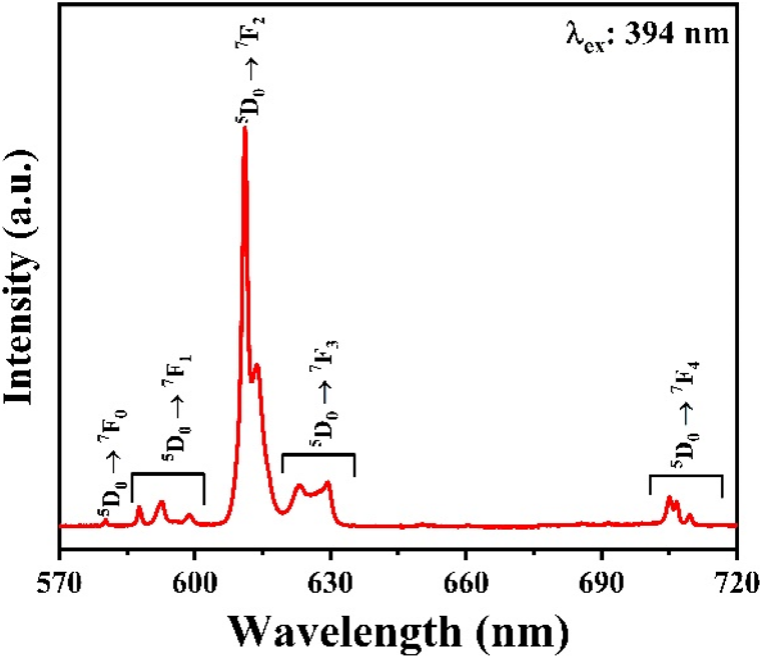
Fluorescence spectrum of Gd_2_O_3_:8% Eu^3+^/CTAB.

To study the optical properties of the Gd_2_O_3_:Eu^3+^/CTAB material, we measured the fluorescence spectrum of the Gd_2_O_3_:Eu^3+^/CTAB sample doped with 8% Eu^3+^ ions. The results obtained in Fig. 7 show that the material emits strongly in the red light region with characteristic emission peaks of Eu^3+^ ions corresponding to the transition from the ^5^D_0_ state to the ^7^F_*J*_ state (*J* = 0–4): ^5^D_0_ → ^7^F_0_ (580 nm), ^5^D_0_ → ^7^F_1_ (588–600 nm), ^5^D_0_ → ^7^F_2_ (607–620 nm), ^5^D_0_ → ^7^F_3_ (620–632 nm) and ^5^D_0_ → ^7^F_4_ (701–712 nm) in which the emission peak at 611 nm has the most vigorous intensity.^27–29^ This emission peak is a transition from the ^5^D_0_ state to the ^7^F_2_ state, where the parity rule does not forbid the electric dipole state of the Eu^3+^ ion. The characteristic properties of this fluorescence spectrum once again demonstrate that the Gd_2_O_3_ matrix has a cubic structure.

**Fig. 8:**
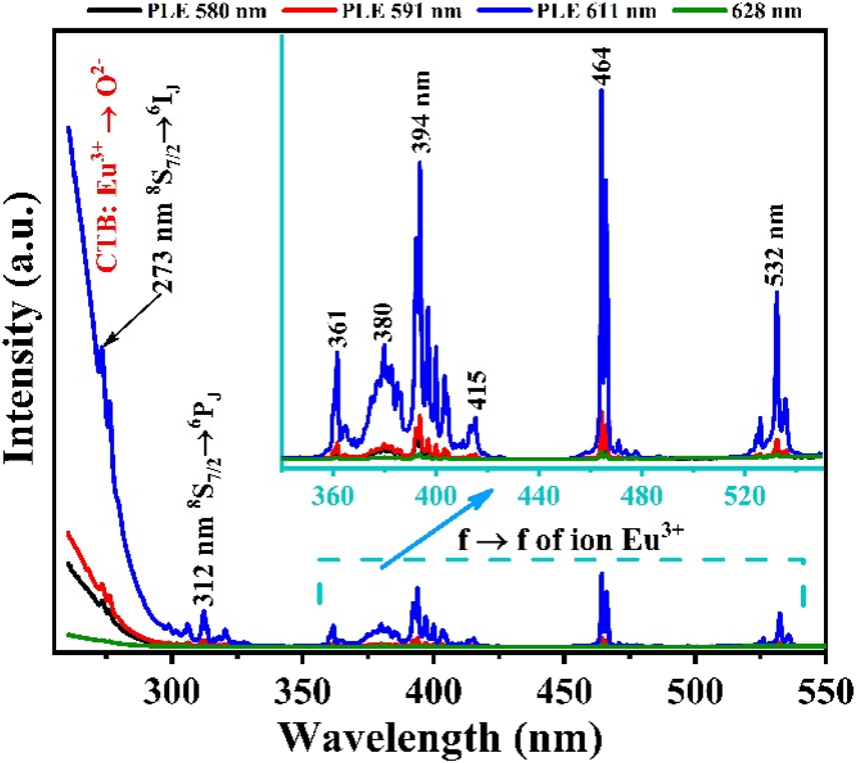
Fluorescence excitation spectrum of Gd_2_O_3_:8% Eu^3+^/CTAB with different emission peaks.

Based on the fluorescence analysis results of the material, we measured the fluorescence excitation spectrum of the Gd_2_O_3_/CTAB material sample doped with 8% Eu^3+^ ions with different emission peaks at 580, 591, 611, and 628 nm. The results obtained in Fig. 8 show that the material strongly absorbs in the ultraviolet and visible regions with excitation peaks at 252, 273, 312, 360, 395, 464, and 532 nm. The strong absorption band in the UV region at 252 nm is attributed to the charge transfer transition (CTB) between Eu^3+^ → O^2−^. The absorption peaks at 273 nm and 312 nm are the characteristic absorption peaks of Gd^3+^ ions corresponding to the ^8^S → ^6^I and ^8^S → ^6^P transitions, respectively.^27–29^ Meanwhile, the excitation peaks at 360, 394, 464, and 532 nm are the characteristic absorption peaks of Eu^3+^ ions corresponding to the f–f transition. Among these absorption bands, the CTB band between Eu^3+^ → O^2−^ is the strongest, and the emission peak for fluorescence excitation has the highest intensity at 611 nm. This result shows that when excited at different wavelengths, the energy level transition of Eu^3+^ ions from the ^5^D_0_ state to the ^7^F_2_ state has the highest transition probability.

To evaluate the emission ability of the material with different excitation wavelengths obtained in Fig. 8. We measured the fluorescence spectrum of Gd_2_O_3_:8% Eu^3+^/CTAB with excitation wavelengths of 273, 394, 464, and 512 nm.

**Fig. 9:**
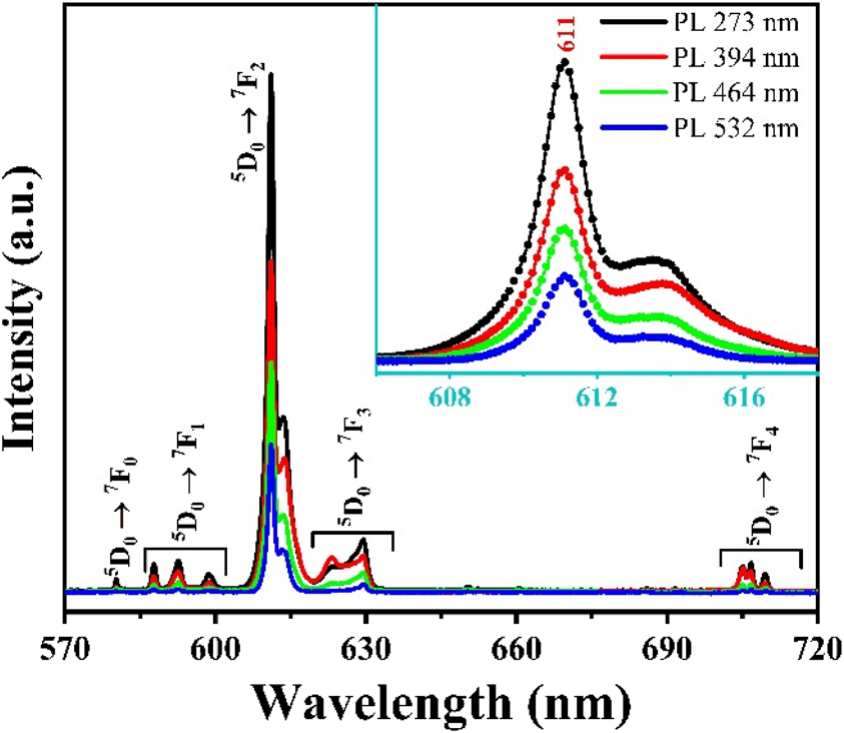
Fluorescence excitation of Gd_2_O_3_:8% Eu^3+^/CTAB.

**Fig. 10:**
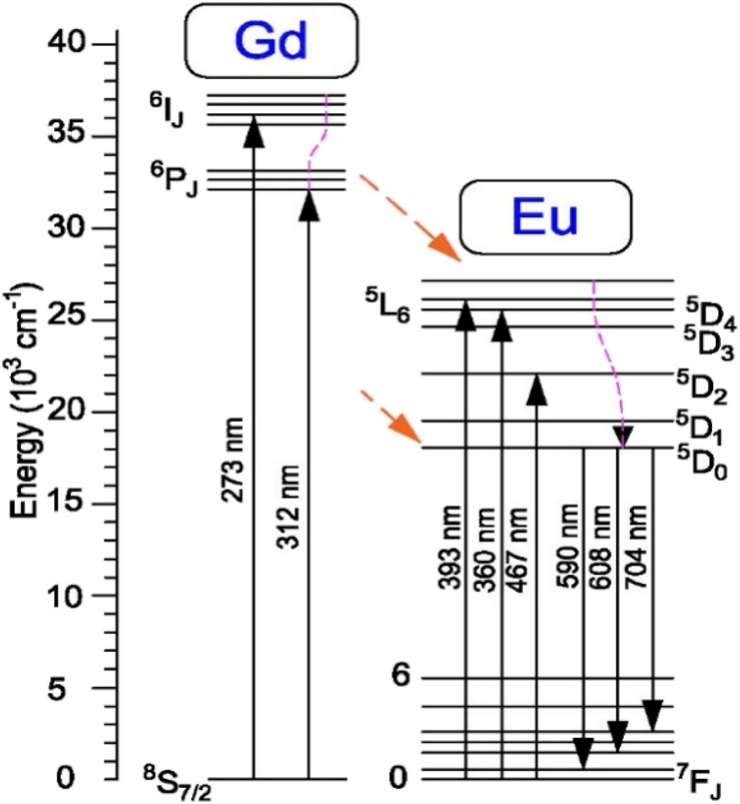
The simplified energy level diagram of Gd^3+^ and Eu^3+^ ions.

The results obtained in Fig. 9 show that the positions of the characteristic emission peaks of Eu^3+^ ions in the Gd_2_O_3_ matrix do not change, but only the intensity of the peaks changes, and no strange peaks are emitted when excited at different wavelengths. This shows that the emission process of the material only includes the energy level transitions of Eu^3+^ ions from the excited state ^5^D_0_ to the state ^7^F_*J*_ without including the emission process of Gd^3+^ ions. With this different excitation wavelength, the material emits best when excited at 273 nm (corresponding to the energy level transition of Gd^3+^ ion from ^8^S–^6^I state), followed by 394 nm. This shows that the absorption process of the Gd_2_O_3_ matrix and Gd^3+^, Eu^3+^ ions, when moving to high energy excited states, all tend to shift without emission to the ^5^D_0_ state of Eu^3+^ ion before shifting to the ^7^F_*J*_ state for characteristic emission of Eu^3+^ ion. The energy transfer process from the ^8^S excited state of Gd^3+^ ion to Eu^3+^ ion does not lead to an emission process that is worth studying. The mechanism of these energy transfer processes is shown in Fig. 10.

With the results of this study, we will investigate the effect of Eu^3+^ doping concentration on the energy transfer mechanism between Gd^3+^ ions and Eu^3+^ ions by measuring the fluorescence spectrum depending on Eu concentration with an excitation wavelength of 273 nm.

**Fig. 11:**
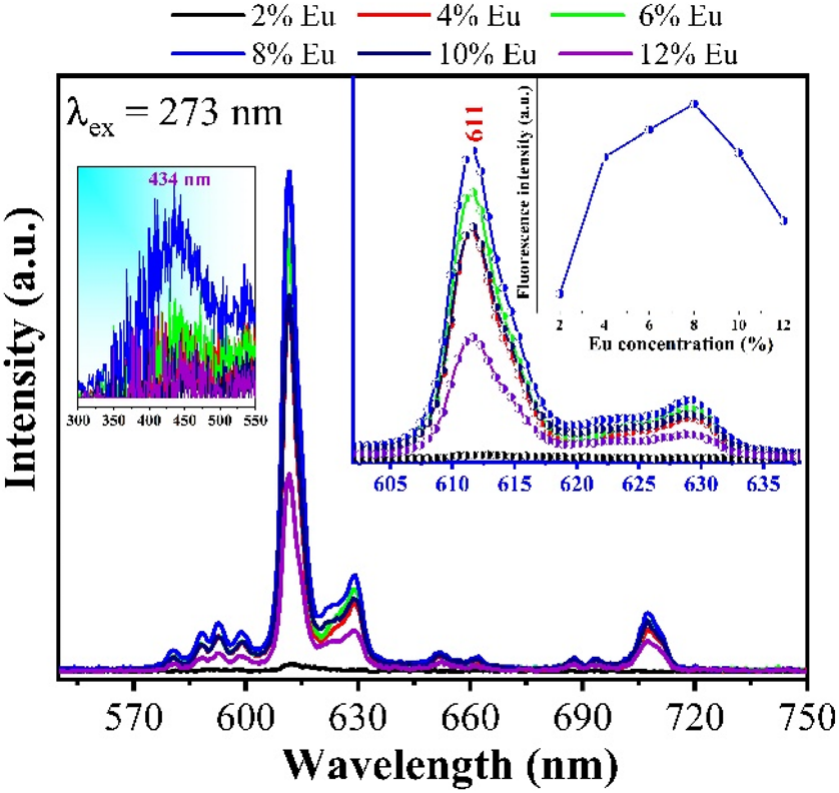
Fluorescence spectra of Gd_2_O_3_:Eu^3+^/CTAB at different concentrations with an excitation wavelength of 273 nm, measured at room temperature.

The analysis results in Fig. 11, when excited at 273 nm with Gd_2_O_3_/CTAB samples doped with Eu^3+^ ions at concentrations from 2 to 12%, show that when the Eu^3+^ ion concentration is at 2%, the intensity of the fluorescence peaks is very low. This shows that with low Eu concentrations, when excited at 273 nm, the absorbed electrons move to the ^6^I_7/2_ state and recombine without emission. Theoretically, Gd^3+^ ions can transfer energy to Eu^3+^ ions when absorbed to a high energy level. However, in the fluorescence spectra of Gd_2_O_3_/CTAB samples doped with Eu^3+^ ions at different concentrations, we did not observe the emission band of Gd^3+^ ions at the 312 nm emission peak corresponding to the transition of the ^6^P excited state to the ^8^S state. This result can be explained by the fact that the Gd^3+^ ion has a stable electron structure in the ^4^f_7_ electron configuration.^30^ Therefore, the host environment has almost no effect on the energy level of Gd^3+^ ions. In addition, considering the very high excitation energy of Gd^3+^ ion, the instantaneous energy transfer from Gd^3+^ ion to Eu^3+^ ion is almost impossible in Gd_2_O_3_/CTAB material doped with low concentration Eu^3+^ ion. This leads to the lifetime of Gd_2_O_3_/CTAB samples doped with low-concentration Eu^3+^ ions being usually longer than that of high-concentration doped samples. When the Eu concentration increases, the charge transfer process between Gd^3+^ ions and Eu^3+^ ions increases, causing the fluorescence intensity in the emission band of Eu^3+^ ions from the ^5^D_0_ state to the ^7^F_*J*_ state to increase and reach a maximum value at a doping concentration of 8% Eu. The fluorescence quenching phenomenon occurs when the doping concentration increases above 8% in the cubic structure of Gd_2_O_3_ nanoparticles. This fluorescence quenching result at high Eu concentration is attributed to the fact that Eu^3+^ ion has a similar radius to Gd^3+^ ion, and at the same time, Gd^3+^ ion acts as a photobleach that enhances the luminescence of Eu^3+^ ion when replacing Gd^3+^ ion in general substrate lattices. The concentration of Eu^3+^ ion doping in this Gd_2_O_3_ substrate lattice is much higher than that in some other substrate lattices such as Sr_6_P_5_BO_20_,^31^ Sr_5_(PO_4_)_3_Cl,^32^ or with monoclinic Gd_2_O_3_ structure.

**Fig. 12:**
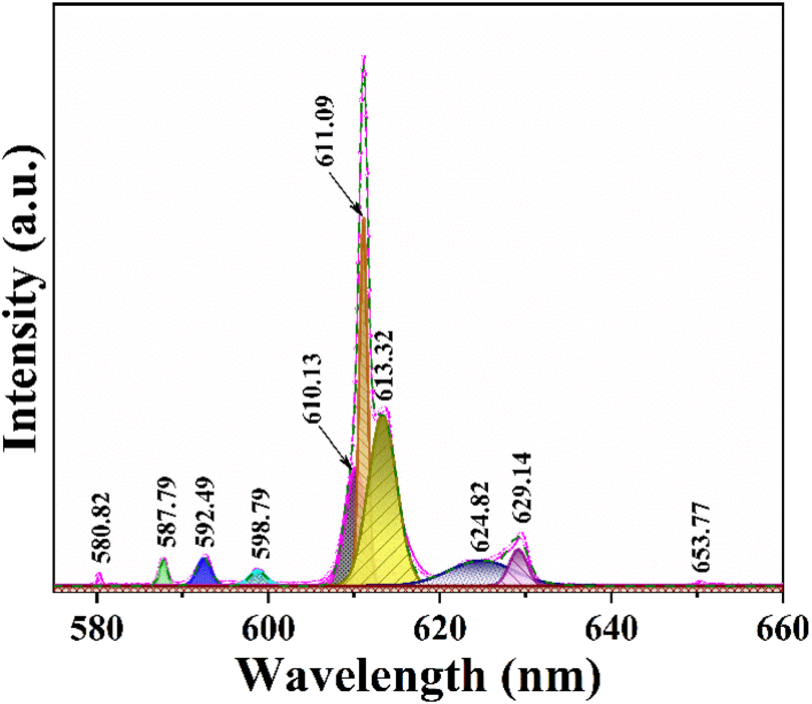
Fluorescence spectrum of Gd_2_O_3_:8% Eu^3+^/CTAB excited at 273 nm fits at different peak positions.

To investigate the crystal symmetry, we analyzed peak splitting in the Gd_2_O_3_:8% Eu^3+^ sample (Fig. 12). The transition from ^5^D_0_ to ^7^F_1_ is identified as an electric dipole transition that is unaffected by local crystal field symmetry. In cubic Gd_2_O_3_, two symmetry positions exist: *C*_2_ and *S*_6_, occurring in a ratio of 3 : 1. The *C*_2_ position lacks inversion symmetry, while the *S*_6_ position possesses it. When Eu ions occupy the *C*_2_ positions, electric dipole transitions from ^5^D_0_ to ^7^F_2_ follow the selection rule Δ*J* = 2. In contrast, when occupying *S*_6_ positions with inversion symmetry, the electric dipole transition from ^5^D_0_ to ^7^F_1_ adheres to Δ*J* = 1.^14^ To determine this issue, we have fitted the fluorescence spectra of the Gd_2_O_3_:8% Eu^3+^ sample at different emission peak positions.

Between 575 nm and 660 nm, several prominent peaks are observed in the luminescence spectrum of Gd_2_O_3_ samples doped with Eu^3+^. These peaks correspond to transitions between energy states, specifically from ^5^D_0_ to ^7^F_0_, ^5^D_0_ to ^7^F_1_, ^5^D_0_ to ^7^F_2_, ^5^D_0_ to ^7^F_3_, and ^5^D_0_ to ^7^F_4_. Here's a detailed explanation of the key transitions:

• ^5^D_0_ to ^7^F_1_ transition (around 587.79, 592.49, and 598.77 nm): in highly symmetric crystal fields, the electric dipole transition from *J* = 1 typically does not split into further sublevels. However, in these samples with a monoclinic crystal structure and reduced symmetry, the *J* = 1 state of ^7^F_1_ splits into three sublevels. This splitting is likely influenced by the concentration of impurities.

• ^5^D_0_ to ^7^F_2_ transition (around 610.13, 611.09, 613.32, 624.82, and 629.14 nm): the characteristic red luminescence of Eu^3+^ arises from this transition, typically occurring between 610 and 630 nm. Due to the *C*_s_ monoclinic symmetry positions of Eu^3+^, the *J* = 2 state of ^7^F_2_ splits into five sublevels. These sublevels are represented by five distinct peaks, indicating transitions from ^5^D_0_ to these ^7^F_2_ sublevels.

To compare the difference between the energy transfer mechanism from Gd^3+^ ions to Eu^3+^ ions, we also investigated the emission ability of Gd_2_O_3_/CTAB samples doped with Eu^3+^ at an excitation wavelength of 394 nm, corresponding to the preferential transition of Eu^3+^ ions from the ground state ^7^F_0_ to the state ^5^L_6_.

**Fig. 13:**
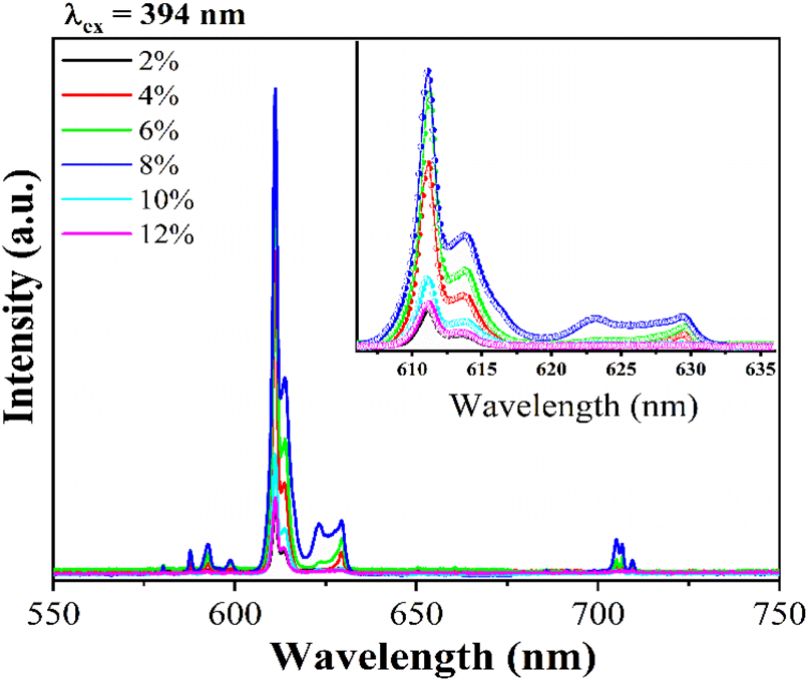
Fluorescence spectra of Gd_2_O_3_:Eu^3+^/CTAB at different concentrations with an excitation wavelength of 394 nm, measured at room temperature.

The results shown in Fig. 13 show that the material emits strongly in the red light region with the position of the characteristic emission peaks of Eu^3+^ ions from the excited state ^5^D_0_ to the state ^7^F_*J*_ almost unchanged compared to when excited at 273 nm. However, we observed that with the 2% doped sample, the intensity of the 611 nm emission peak is relatively large compared to this sample when excited at 273 nm.

This result indicates that, for the energy transfer phenomenon between Gd^3+^ ions and Eu^3+^ ions to occur, the Eu concentration must be large enough to receive this energy transfer process. To compare the above results, we have established the ratio between the 611 nm peak intensity of samples with different concentrations, with the 8% doped Gd_2_O_3_/CTAB sample giving the most vigorous intensity; the results are shown in Table 1.

**Table 1:** Intensity ratio *I*_*x*_/*I*_0_ (%) (*I*_0_ is the 611 nm peak intensity of the Gd_2_O_3_/CTAB sample doped with 8% Eu^3+^ ions)

Eu^3+^ concentration	Excitation 273 nm	Excitation 394 nm
2	1.16	14.40
4	52.35	67.08
6	73.57	92.12
8	100	100
10	48.61	25.39
12	33.01	16.23

**Fig. 14:**
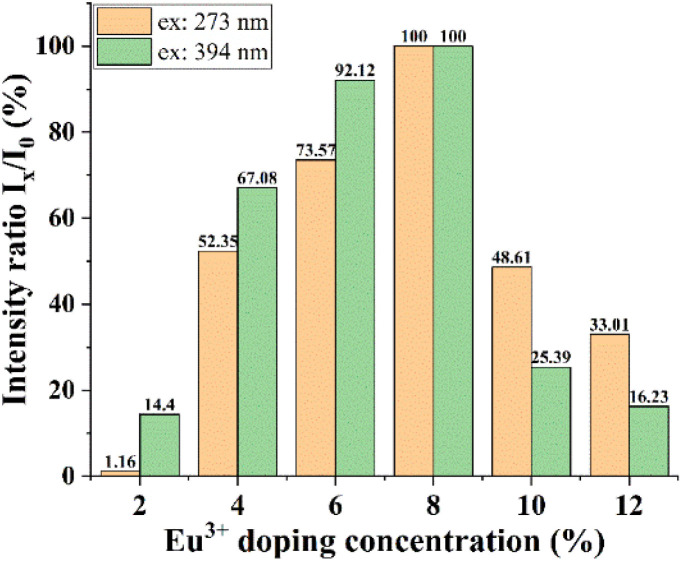
*I*_*x*_/*I*_0_ intensity ratio of Gd_2_O_3_/CTAB samples with different Eu concentrations, with excitation wavelengths of 273 nm and 394 nm.

Based on the results obtained in Table 1, we have drawn a graph showing this intensity ratio in Fig. 14. The analysis results show that with low doping concentrations, the energy transfer process between Gd^3+^ ions and Eu^3+^ ions is more complex, making the intensity ratio of the 611 nm peak of the samples compared to the sample with the highest intensity when excited at 273 nm. At the same time, when excited at 394 nm, the intensity ratio of this 611 nm peak to the sample with the highest intensity is stronger. However, when the phenomenon of fluorescence quenching due to concentration occurs, when excited at 273 nm, the quenching process occurs more slowly. This shows that the energy transfer process from Gd^3+^ ions to Eu^3+^ ions reduces the non-radiative recombination process in the material when the doping concentration is high. To supplement this study, we measured the time-resolved fluorescence spectra of Gd_2_O_3_/CTAB samples doped with Eu^3+^ ions with different doping concentrations, with an excitation wavelength of 273 nm and an emission wavelength of 611 nm.

### 3.4. Effect of Eu concentration on PL lifetime

**Fig. 15:**
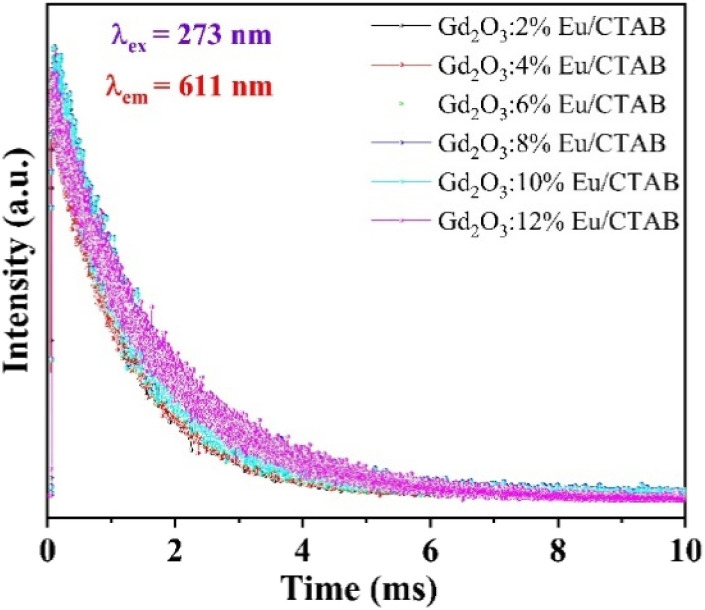
Decay curves of Gd_2_O_3_:Eu^3+^/CTAB with different mol concentrations.

To investigate the influence of the concentration of Eu^3+^ ions doped into the matrix of the material, we also measured the time-resolved fluorescence spectra of Gd_2_O_3_/CTAB samples doped with Eu^3+^ ions with doping concentrations from 2 to 12% corresponding to the fluorescence excitation peak at 273 nm and the emission peak at 611 nm (Fig. 15) The results showed that the curves fit a quadratic, exponential function, indicating two separate origins of the emission process in Gd_2_O_3_/CTAB materials doped with Eu^3+^ ions eqn (3):3
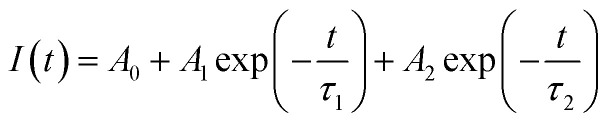
where *t* is the time, *I*(*t*) is the luminescence intensity at time *t*, *A*_1_ and *A*_2_ are constants, and *τ*_1_, *τ*_2_ are exponential components of the decay time. The value of the average lifetime τ* can be calculated using the following formula eqn (3) and (4):4
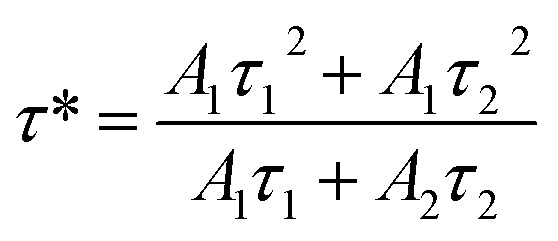


Table 2 shows the average lifetime of Gd_2_O_3_:*x*% Eu^3+^/CTAB (*x* = 2–12%) samples calculated by formula (3).

**Table 2:** Average lifetimes of Gd_2_O_3_:*x*% Eu^3+^/CTAB (*x* = 2–12%) phosphors excited at 273 nm and monitored at 611 nm

Eu^3+^ concentration	Average lifetime, τ* (ms)	Chromaticity diagram (*x*, *y*)	
2	1.18	0.61	0.31
4	1.29	0.62	0.34
6	1.36	0.59	0.33
8	2.1	0.64	0.34
10	1.42	0.63	0.33
12	1.38	0.64	0.33

With the results of fluorescence lifetime analysis of the materials listed in Table 2, it can be seen that, as the Eu concentration increases, the average lifetime of electrons in the excited state tends to increase from 1.18 ms with the 2% Eu doped sample and reaches a maximum of 2.1 ms with the 8% Eu doped Gd_2_O_3_/CTAB sample and then tends to decrease. The results of the lifetime analysis of these Eu-doped Gd_2_O_3_/CTAB samples with different concentrations are consistent with the fluorescence analysis results in Section 3.3, as we have analyzed the energy transfer mechanism shown in Fig. 10. The energy transfer mechanisms in Eu^3+^-doped Gd_2_O_3_/CTAB materials when the material is excited with a wavelength of 273 nm can include: T1(O^2−^ → Eu^3+^) related to the direct energy transfer between the CTB band to the Eu^3+^ ions; T2(Gd^3+^ → Eu^3+^) related to the energy transfer between the Gd^3+^ ions in the excited state ^6^I_*J*_ to the Eu^3+^ ions; T3(Eu^3+^ → Eu^3+^) related to the direct energy transfer of the Eu^3+^ ions in the excited state to each other. This is one of the most important mechanisms for concentration-dependent fluorescence quenching. Finally, T4(Eu^3+^ → O^2−^) is the back transfer between Eu^3+^ ions to O^2−^; in this process, the back transfer of energy of Eu^3+^ ions from the excited state to the CTB band. During the fluorescence excitation process at 273 nm, the T1 transition is limited because this energy is only enough to excite Gd^3+^ ions to the ^6^I_*J*_ state. With low-concentration Eu^3+^ doped Gd_2_O_3_/CTAB samples, the T2 process is less likely to occur than with high-concentration samples because the density of Eu^3+^ ion emission centers in the material is low, so the energy transfer phenomenon between Gd^3+^ ions in the ^6^I_*J*_ excited state is less likely to occur. We analyzed this result in Section 3.3 when comparing the fluorescence intensity of this sample with the sample with the highest intensity, which is only 1.16%.

Meanwhile, when excited at 394 nm, this ratio is 14.40%. When the Eu doping concentration increases, the density of the emission center increases, this process increases the non-radiative recombination when the Gd^3+^ ion transfers energy from the excited state ^6^I_*J*_ to the excited state ^5^D_*J*_ of the Eu^3+^ ion, increasing the lifetime of electrons in the excited state, while increasing the electron density in the ^5^D_0_ state. This process increases the transition of electrons in the ^5^D_0_ state to the ^7^F_*J*_ state, increasing the fluorescence intensity when the concentration increases. When the Eu concentration increases, the T3 energy level transfer process increases, reducing the electron density in the excited state by the reverse energy transfer process between Eu^3+^ ions, reducing the fluorescence lifetime and intensity due to concentration-dependent fluorescence quenching. The results of the fluorescence lifetime analysis of the material with an excitation wavelength of 273 nm, with an emission peak of 611 nm, are very consistent with the fluorescence analysis results developed in Section 3.3.

### 3.5. Calculation of Judd–Ofelt parameters

The probability of electric dipole transitions from the ^5^D_0_ state to the ^7^F_*J*_ state (where *J* = 2, 4, 6) is determined by the following formula eqn (5):^19^5
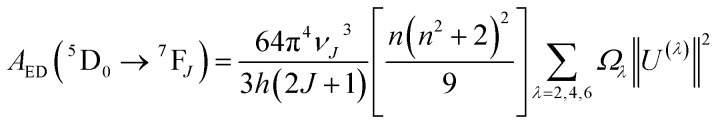
Here, *ν*_*J*_ represents the energy of the ^5^D_0_ to ^7^F_*J*_ transition, *e* is the electron charge, and ‖*U*^(*λ*)^‖^2^ angle denotes the reduced matrix element of the unit tensor operator of rank *λ* = 2, 4, 6. The Judd–Ofelt parameter *Ω*_*λ*_ can be calculated by analyzing the intensity ratios of the ^5^D_0_ to ^7^F_*J*_ transitions for *J* = 2, 4, 6 as follows eqn (6):6
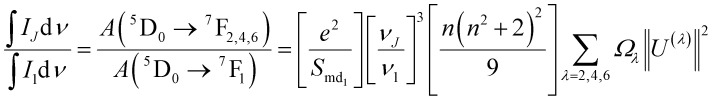


For the transitions ^5^D_0_ → ^7^F_2_, the matrix elements are *U*^(2)^ = 0.0033, *U*^(4)^ = *U*^(6)^ = 0. For the ^5^D_0_ → ^7^F_4_ transitions, *U*^(2)^ = 0, *U*^(4)^ = 0.0023, and *U*^(6)^ = 0. Lastly, for the ^5^D_0_ to ^7^F_6_ transitions, *U*^(2)^ = *U*^(4)^ = 0 and *U*^(6)^ = 0.003. The total area of the absorption bands for ^5^D_0_ to ^7^F_*J*_ (with *J* = 2, 4, 6) and ^5^D_0_ → ^7^F_1_ is also considered.

The intensity parameters *Ω*_*λ*_ provide valuable insights into the local environment surrounding the Eu^3+^ ion. The parameter *Ω*_2_ is particularly sensitive to changes in ligand asymmetry and the covalency of the Eu^3+^–ligand bond: a high *Ω*_2_ value indicates significant ligand asymmetry and high covalency in the Eu^3+^–ligand bond. On the other hand. *Ω*_4_ reflects the rigidity of the environment embedding the rare-earth ion; a high *Ω*_4_ value corresponds to lower environmental rigidity (Table 3).^18^

**Table 3:** Calculation of *Ω*_2,4,6_ parameters for Gd_2_O_3_:Eu^3+^ at various molar concentrations

Samples	*Ω*_2_ (×10^−20^ cm^2^)	*Ω*_4_ (×10^−20^ cm^2^)	*Ω*_6_ (×10^−20^ cm^2^)
Gd_2_O_3_:2% Eu^3+^	6.63	1.15	0
Gd_2_O_3_:4% Eu^3+^	8.1	3.22	0
Gd_2_O_3_:6% Eu^3+^	12.2	3.29	0
Gd_2_O_3_:8% Eu^3+^	14.2	4.01	0
Gd_2_O_3_:10% Eu^3+^	11.2	3.94	0
Gd_2_O_3_:12% Eu^3+^	11	3.95	0

The transition probability from the excited state *J* to a lower state *J*′ determines the fluorescence intensity of the *J* to *J*′ transition eqn (7):7
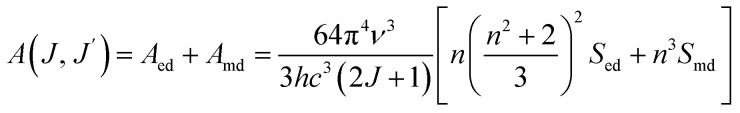


Total transition probability and lifetime of the excited state *J*eqn (8) and (9):8
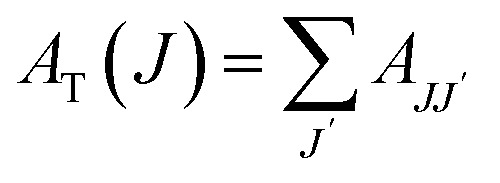
9
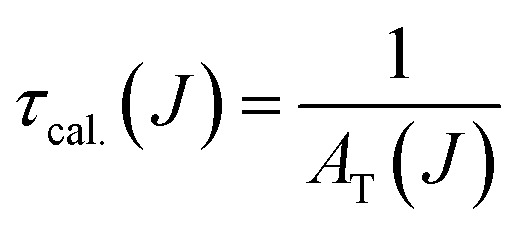


Branching ratio: used to predict the relative intensity of a fluorescence band from an excited state. The theoretical branching ratio is calculated using the formula eqn (10):10
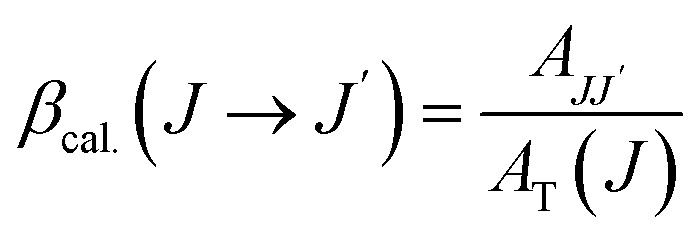


Quantum efficiency is determined using the following formula eqn (11):11
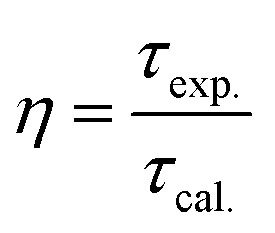


Quantum efficiency calculations of Eu-doped Gd_2_O_3_/CTAB materials show that the quantum efficiency ranges from 40% to 91% depending on the Eu doping concentration. The sample with the lowest quantum efficiency is the Gd_2_O_3_/CTAB sample doped with 2% Eu^3+^ ions; the quantum efficiency gradually increases and reaches a maximum value of 91% with the Gd_2_O_3_:8% Eu^3+^ sample and tends to decrease when the doping concentration is above 8%. The results obtained based on the theoretical model are consistent with the fluorescence survey results. When the Eu concentration is low, the energy transfer phenomenon between Gd^3+^ ions and Eu^3+^ ions is more challenging, so the material absorbs and leads to large non-radiative recombination. When the concentration of Eu^3+^ ions increases, the density of emission centers increases, and the energy transfer process between Gd^3+^ ions and Eu^3+^ ions is more effective, so the quantum efficiency increases. When the doping concentration reaches 8%, the fluorescence quenching phenomenon occurs, so the quantum efficiency tends to decrease. The result obtained for the highest quantum efficiency is 91%, which is larger than the result we obtained.^33^ When synthesizing this material in the environment, only urea solution and TEOS or TOPO were used (Table 4).

**Table 4:** Branching ratio parameters, lifetime, and quantum efficiency of Gd_2_O_3_:Eu^3+^/CTAB at various molar concentrations

Samples	*β*_exp._ (%)	*β*_cal._ (%)	*A*_T_	*τ*_cal._ (ms)	*τ*_exp._ (ms)	*η* (%)
Gd_2_O_3_:2% Eu^3+^	76.2	76	334.569	2.9	1.18	40.1
Gd_2_O_3_:4% Eu^3+^	80.1	80	587.111	1.7	1.29	75.8
Gd_2_O_3_:6% Eu^3+^	76.5	75	678.170	1.5	1.36	90.1
Gd_2_O_3_:8% Eu^3+^	76.5	72	426.650	2.3	2.1	91
Gd_2_O_3_:10% Eu^3+^	77.2	77	561.172	1.7	1.42	83
Gd_2_O_3_:12% Eu^3+^	75.1	74	527.372	1.8	1.38	76

### 3.6. Magnetic properties

**Fig. 16:**
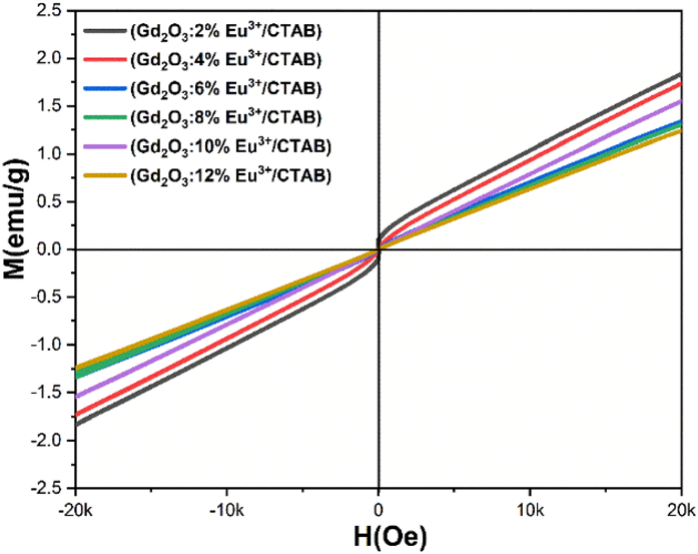
The hysteresis curve of Gd_2_O_3_:Eu^3+^/CTAB for different dopant concentrations.

The magnetic properties of the Gd_2_O_3_:Eu^3+^ material with [Eu^3+^]/[Gd^3+^] ratios of 2, 4, 6, 8, 10, and 12 mol% were analyzed using a vibrating sample magnetometer (VSM) on a MicroSense EZ9 (USA), as shown in Fig. 16. The magnetism of the samples was measured at room temperature under an applied magnetic field of 20 kOe using the VSM system.

The paramagnetic properties of Gd_2_O_3_:Eu^3+^/CTAB arise from the presence of seven unpaired electrons in the 4f shell of Gd^3+^. These unpaired electrons are shielded from the crystal field by the outer 5s^2^5p^6^ shell electrons.^14^ The shape of the hysteresis curve (M–H) varies across all samples with different concentrations of Gd and Eu ions, likely due to changes in the size of the synthesized particles. As shown in Fig. 16, the magnetism of the Gd_2_O_3_ material reaches a peak value of 1.83 emu g^−1^. This value is twice as high as that reported by Zhang *et al.*^34^ and comparable to the results obtained by Xu *et al.*^35^ Although the addition of Eu^3+^ leads to a decrease in magnetic value, the Gd_2_O_3_:12% Eu^3+^ sample exhibits the lowest magnetism at 1.23 emu g^−1^, which aligns with the values reported by Zhang *et al.*^34^ Consequently, Gd_2_O_3_:Eu^3+^/CTAB material demonstrates significant potential for enhancing the contrast in magnetic resonance imaging (MRI).

To evaluate the potential application of the material's magnetothermal effect in targeting cancer cell destruction, we conducted an investigation of the material's heat generation capability. The inductive heating experiment was conducted in an alternating magnetic field with a frequency of 390 kHz and an intensity of 300 Oe. This magnetic field was generated by an induction coil (7 turns, 3 cm in diameter and 11.5 cm long) connected to a commercial RDO-HFI generator with an output power of 5 kW. The magnetic field intensity was calculated using the formula: *H* = *nI*, where *n* is the number of coil turns per unit length, and I is the amplitude of the alternating current flowing through the coil. The samples for measurement were dissolved in a water solution and thermally insulated from the external environment using a vacuum-drawn glass bottle maintained at 10^−3^ to 10^−4^ torr. Temperature readings were taken using an optical thermometer (GaAs sensor, Opsens) with an accuracy of ±0.3 °C within the range of 0 to 250 °C. The specific loss power (SLP) was calculated using the following formula eqn (12):12
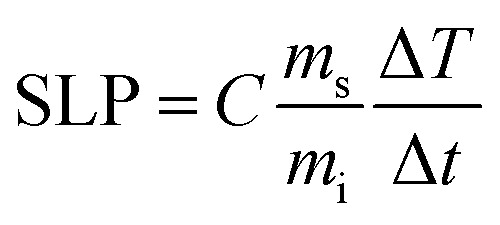
where *C* = 4.18 kJ kg^−1^ K^−1^ represents the specific heat capacity of the sample system (comprising magnetic particles and the solution), *m*_s_ is the total mass of the sample system, and *m*_i_ is the mass of the magnetic particles. The initial rate of temperature increase is determined from the tangent of the temperature–time curve at the moment the magnetic field is activated. For an isolated system, the specific loss power (SLP) value is equivalent to the specific absorption rate (SAR).

**Fig. 17:**
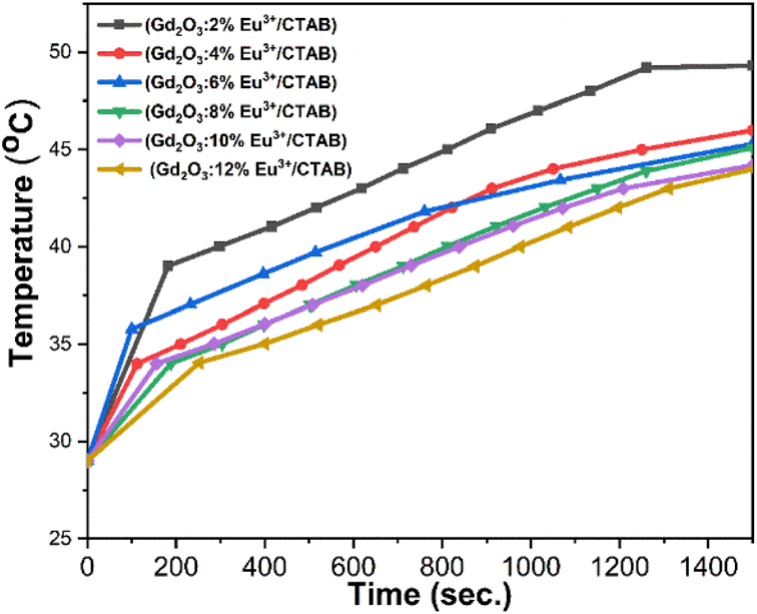
Heating curves of Gd_2_O_3_:Eu^3+^/CTAB at varying dopant concentrations.

The results indicate that the samples achieve temperatures ranging from 44 to 49 °C. Physiological studies on cancer cells have demonstrated their limited heat tolerance, identifying a suitable temperature range of 42 to 49 °C for effectively destroying cancer cells without harming healthy ones.^36^ Therefore, in the research and development of nanomaterials for magnetic heating, it is essential to establish conditions that meet these criteria. With Eu^3+^ doped at molar ratios of 2%, 4%, 6%, 8%, 10%, and 12%, the observed temperatures were 49 °C, 46 °C, 45.3 °C, 45.1 °C, 44.2 °C, and 44 °C, respectively. While increasing the Eu^3+^ doping concentration enhances luminescent properties, it simultaneously reduces the material's magnetic properties. Thus, it is crucial to select an optimal medium doping ratio of Eu^3+^ that balances both luminescent and magnetic properties. These findings confirm that these material systems possess magnetism and can be effectively utilized in magnetic hyperthermia applications (Fig. 17).

To provide a clearer overview, Table 5 presents the parameters from the magnetic induction heating experiment with Gd_2_O_3_:Eu^3+^/CTAB samples at molar ratios of 2%, 4%, 6%, 8%, 10%, and 12%. The table includes magnetic field intensity, saturation temperature at 1500 seconds, initial heating rate, specific absorption power, and material concentration.

**Table 5:** Experimental parameters of magnetic induction heating for Gd_2_O_3_:Eu^3+^/CTAB at varying dopant concentrations

Sample	(*H*, Oe)–(*f*, kHz)	*T*_s_ (°C)	d*T*/d*t* (°C s^−1^)	SAR (W g^−1^)
Gd_2_O_3_:2% Eu^3+^	300 Oe	49.3	0.054	11.29
Gd_2_O_3_:4% Eu^3+^		46.0	0.044	18.40
Gd_2_O_3_:6% Eu^3+^		45.3	0.068	9.20
Gd_2_O_3_:8% Eu^3+^		45.1	0.027	5.53
Gd_2_O_3_:10% Eu^3+^		44.2	0.032	6.69
Gd_2_O_3_:12% Eu^3+^		44.0	0.020	4.18

The specific absorption rate (SAR) values for Gd_2_O_3_ doped with Eu^3+^ at various molar concentrations in the presence of CTAB reveal that SAR increases at lower doping concentrations, while it gradually decreases at higher concentrations. This trend indicates that an increase in the Eu^3+^ ratio within the composite nanoparticles leads to a reduction in the magnetization saturation (MS) value, which subsequently decreases heat generation from magnetic induction. However, the SAR remains adequate to achieve temperatures above 42 °C, ensuring its suitability for magnetic induction heating applications.

## 4. Conclusions

In this study, Gd_2_O_3_:Eu^3+^/CTAB material was synthesized by chemical method through many steps in the presence of CTAB. The obtained material has a spherical shape, an average size distribution from 40 to 220 nm, and a typical cubic structure of Gd_2_O_3_. The material's band gap depends on the doping concentration of Eu^3+^ ions. The band gap tends to decrease when the Eu doping ratio increases and reaches the smallest value of about 3.44 eV when the doping ratio is 8%. Then, the band gap tends to increase when the concentration increases above 8%. This result is because when the Eu concentration increases, Eu^3+^ ions replace Gd^3+^ ions in the matrix of the material, forming emission centers in the band gap of the Gd_2_O_3_ material, causing the band gap to decrease. When the Eu ratio increases, these ions tend to cluster together and escape from the Gd_2_O_3_ material's matrix, causing the material's band gap to increase when the doping ratio increases above 8%.

The Gd_2_O_3_:Eu^3+^/CTAB material strongly absorbs in the ultraviolet region, giving strong emission in the red light region with characteristic emissions of Eu^3+^ ions from the ^5^D_0_ excited state to the ^7^F_*J*_ state (*J* = 0–4). The fluorescence spectrum shows that when the Eu doping ratio is low, the energy transfer process between the bright Gd^3+^ ions and the Eu^3+^ ions is low. The fluorescence quenching phenomenon due to the concentration of this material system corresponds to the doping ratio of 8%. With the best-emitting sample, Gd_2_O_3_:Eu^3+^/CTAB has a quantum efficiency of about 91%.

In addition, the Gd_2_O_3_:Eu^3+^/CTAB material also exhibits weak ferromagnetic properties, with a maximum magnetic field of 1.83 emu g^−1^. This result is twice as high as the maximum magnetic field obtained by other groups using different precursors when synthesizing the material instead of CTAB. Magneto-thermal experiments show that the material can reach temperatures from 43 to 49 °C, within the optimal range for effectively killing cancer cells without harming surrounding healthy cells.

Overall, the results obtained for the Eu^3+^ ion-doped Gd_2_O_3_/CTAB material show that it possesses optical and magnetic properties, making it a promising candidate for multifunctional applications in diagnosis and treatment.

## Data availability

All data are presented in the article.

## Author contributions

Pham Thi Lien: writing – original draft, methodology, investigation, formal analysis, data curation, conceptualization. Tong Quang Cong: methodology, investigation. Vu Ngoc Phan: methodology, investigation. Vu Thi Hong Ha: writing – original draft, methodology. Ho Thi Anh: methodology, investigation. Pham Duc Thang: methodology, investigation, Le Thi Hoi: methodology, investigation. Dinh Manh Tien: methodology, formal analysis. Nguyen Thanh Huong: methodology, investigation. Hoang Thi Khuyen: methodology, investigation. Lam Thi Kieu Giang: methodology, investigation. Nguyen Vu: methodology, formal analysis. Pham Hong Nam: methodology, formal analysis. Le Tien Ha: writing – review & editing, and editing the final manuscript.

## Conflicts of interest

The authors declare that they have no known competing financial interests or personal relationships that could have appeared to influence the work reported in this paper.
